# Eupædia; or, Letters to a Mother on the Watchful Care of Her Infant, in Reference to Diet, Clothing, Air, Exercise, Medicine, &c.

**Published:** 1836-07

**Authors:** 


Art. XV.—*Eupædia; or, Letters to a Mother on the watchful Care of her Infant, in reference to Diet, Clothing, Air, Exercise, Medicine, &c.* By a Physician.—*London*, 1836. 18mo. pp. 144.

We have read this little book with much pleasure, and we can conscientiously recommend it to our readers as one of the very few of a medical character that can be put into the hands of non-professional persons, not only with safety but advantage. While suited to the capacity and understanding of every well-educated mother, it is entirely free from all absurdities either of theory or practice; and as it professes only to teach the knowledge of diseases so far as to guard against them, and to recognise their approach, with the view to the summoning of the only legitimate prescriber, or, at most, enjoins the provisional employment of simple and innocuous remedies, it is to be regarded as entirely distinct from the class of "Guides" and "Domestic Medicines" for the nursery. Although inferior both in its plan and its execution to the admirable "Advice to young Mothers, by a Grandmother," published many years since, it is in some respects more suited to the purpose which it professes, of giving advice to mothers which they can understand, and can follow without risk of danger.

In going over the pages we marked several passages both for commendation and criticism; and we must say, that the number of the former greatly exceeded the latter. Indeed, with the exception of a frequently too fine or too ambitious style, there were only two or three points in which we did not entirely concur with the author. We were a little startled at finding the mother counselled to study and practise *auscultation* (Letter VI.,) with the view of being in a condition to give full information to the physician; and we apprehend it will be some time before this advice can be followed with advantage. We were also (perhaps unjustly) startled at the zealous recommendation to the mother to learn "*to lance the little patient's gums herself*," (p. 106-107), since such a practice certainly is likely to be much less injurious than the administration of many powerful internal medicines which mothers now habitually give. Of the additional direction (p. 133,) that, "when the gums have once been lanced, they may be prevented from closing, by passing a common ivory knitting needle down to the teeth or socket *daily*," we cannot possibly approve, whether the operator be lay or professional. We also strongly object to the following piece of advice addressed to the mother (nurse): "meat should be taken *three times daily*, in moderate quantities each time. It is often necessary to add ale or porter, and other nutritious fluid." (P. 62.) Surely the author cannot mean that all nurses should eat meat (i. e. animal food) thrice daily, whether they have been accustomed to it or not? Most certainly such a practice would tend to disorder, and thereby debilitate the system, in the case of delicate mothers, accustomed at other times to a quite different regimen. Indeed, we do not hesitate to say that infinitely more mischief is done to the health of nurses by the opinion, universally prevalent among women, that it is necessary to eat and drink a great deal, "in order to keep up their strength," than by any other cause; and we are surprised to find the author of this little work, (who, we have reason to believe, is really a physician of great experience,) sanctioning such a prejudice—for such we must call it—by his high authority. We are the more surprised at this, as we find, among the passages noted for commendation, more than one relating to the dieting of the child, all of which bear strongly on the importance of simplicity and moderation in this particular. How just are the following observations:

"The great error committed in regard to the diet of infants, is not so much that of improper *kinds* of food as of its undue *quantity*." (P. 68.) "The stomach, even of an infant, will bear a little improper food with greater impunity than too much even of that which is most suitable. I believe few infants, if any, among the rich have suffered from too little food, but an innumerable number have paid with their lives the penalty of over-feeding." (P. 70.)

We conclude by quoting a passage, with which we ourselves are still more pleased, and which we recommend to the consideration of all our readers who are themselves parents, as no less philosophical than practically true.

"During this daily process of washing, which should not be done languidly but briskly and expeditiously, the mind of the little infant should be amused and excited. In this manner dressing, instead of being dreaded, as a period of daily suffering,—instead of being painful, and one continued fit of crying, will become a recreation and amusement. In this, treat your infant, even your little infant, as a sensitive and intelligent creature. Let every thing which *must* be done, be made not a source of pain, but of pleasure, and it will then become a source of health, and that both of body and mind; a source of exercise to the one, and of early discipline to the other. Even at this tender age, the little creature may be taught to be patient, and even gay, under suffering. Let it be remembered that every act of the nurse towards the little infant, is productive of good or evil upon its character as well as health. Even the acts of washing and clothing may be made to discipline and improve the temper, or to try and impair it, and may therefore be very influential on its happiness in future life. For thus it may be taught to endure affliction with patience and even cheerfulness, instead of fretfulness and repining. And every infliction upon the temper, is also an infliction upon the body and the health of the little child. The parent and the nurse should, therefore, endeavour to throw her own mind into her duties towards her offspring. And in her intention of controlling her infant's temper, let her not forget that the first step is to control her own. How often have I observed an unhappy mother the parent of unhappy children!" (P. 89.)

